# Nutritional Support and Physical Modalities for People with Osteoporosis: Current Opinion

**DOI:** 10.3390/nu11122848

**Published:** 2019-11-20

**Authors:** Li-Ru Chen, Peng-Hsuan Hou, Kuo-Hu Chen

**Affiliations:** 1 Department of Physical Medicine and Rehabilitation, Mackay Memorial Hospital, Taipei 10449, Taiwan; gracealex168@gmail.com (L.-R.C.); u9702455@cmu.edu.tw (P.-H.H.); 2 Department of Mechanical Engineering, National Chiao-Tung University, Hsinchu 300, Taiwan; 3 Department of Obstetrics and Gynecology, Taipei Tzu-Chi Hospital, The Buddhist Tzu-Chi Medical Foundation, Taipei 23142, Taiwan; 4 School of Medicine, Tzu-Chi University, Hualien 970, Taiwan

**Keywords:** osteoporosis, calcium, vitamin D, exercise, physical modality

## Abstract

Osteoporosis is a vital healthcare issue among elderly people. During the aging process, a gradual loss of bone mass results in osteopenia and osteoporosis. Heritable factors account for 60–80% of optimal bone mineralization, whereas modifiable factors such as nutrition, weight-bearing exercise, body mass, and hormonal milieu affect the development of osteopenia and osteoporosis in adulthood. Osteoporosis substantially increases the risk of skeletal fractures and further morbidity and mortality. The effective prevention of fractures by reducing the loss of bone mass is the primary goal for physicians treating people with osteoporosis. Other than pharmacologic agents, lifestyle adjustment, nutritional support, fall prevention strategies, exercise, and physical modalities can be used to treat osteoporosis or prevent further osteoporotic fracture. Each of these factors, alone or in combination, can be of benefit to people with osteoporosis and should be implemented following a detailed discussion with patients. This review comprises a systematic survey of the current literature on osteoporosis and its nonpharmacologic and nonsurgical treatment. It provides clinicians and healthcare workers with evidence-based information on the assessment and management of osteoporosis. However, numerous issues regarding osteoporosis and its treatment remain unexplored and warrant future investigation.

## 1. Introduction

An increasingly aging population is now a global phenomenon, especially in developed countries [1]. Thus, interventions are needed to ameliorate physical fitness-related changes to guarantee higher functional capacity, autonomy, and health among elderly people.

Osteoporosis is a vital healthcare issue among the elderly people. During the aging process, a gradual loss of bone mass results in osteopenia and osteoporosis. Both sexes are affected; however, the main burden of disease falls upon menopausal women. Osteoporosis substantially increases the risk of skeletal fractures and further morbidity and mortality [2,3]. The cumulative lifetime fracture risk for a 50-year old woman with osteoporosis can be as high as 60% [4]. Effective fracture prevention, if achievable by reducing the loss of bone mass, will have a major impact on an individual’s morbidity and, to a lesser extent, mortality [2].

The diagnostic difference between osteopenia and osteoporosis is based on the level of bone mineral density (BMD). The World Health Organization (WHO) recommends measuring BMD at the spine, hip, or forearm using dual-energy X-ray absorptiometry devices [5]. According to the WHO criteria, osteopenia is defined as a BMD between 1.0 and 2.5 SD below that of a “young normal” adult (T score between −1.0 and −2.5), and osteoporosis as a T score of −2.5 or lower.

Some people may also have a congenitally lower bone density. Childhood and adolescence are important stages in optimal bone formation and thus the prevention of osteoporosis in older age. Although heritable factors account for 60–80% of optimal bone mineralization, modifiable factors such as weight-bearing exercise, nutrition, body mass, and hormonal milieu affect the development of osteopenia and osteoporosis in adulthood [1].

A primary goal in the screening and treating of osteoporosis is to prevent the development of subsequent osteoporotic fractures. Effective fracture prevention will have a major impact on an individual’s morbidity and mortality [2]. BMD changes following treatment might also provide a better prediction of the anti-fracture efficacy of a therapeutic agent.

At a molecular level, bone is predominantly composed of the type 1 collagen matrix, which is strengthened with the apposition of calcium hydroxyapatite crystals [6]. Osteoblasts are derived from bone marrow stromal cells (BMSCs), which are self-renewing in vivo. Osteoclasts are multi- nucleated giant cells that resorb the bone matrix, ensuring development and continuous remodeling of the skeleton and the bone marrow hematopoietic niche [7]. Osteocytes are the most numerous, longest-lived cells of bone. They are osteoblasts that become entombed in lacunae in the bone matrix (osteoid) that they synthesize [6]. These osteoblasts undergo a morphologic change and become osteocytes with cytoplasmic processes that connect them with other osteocytes and flattened lining cells [6].

**Figure 1:**
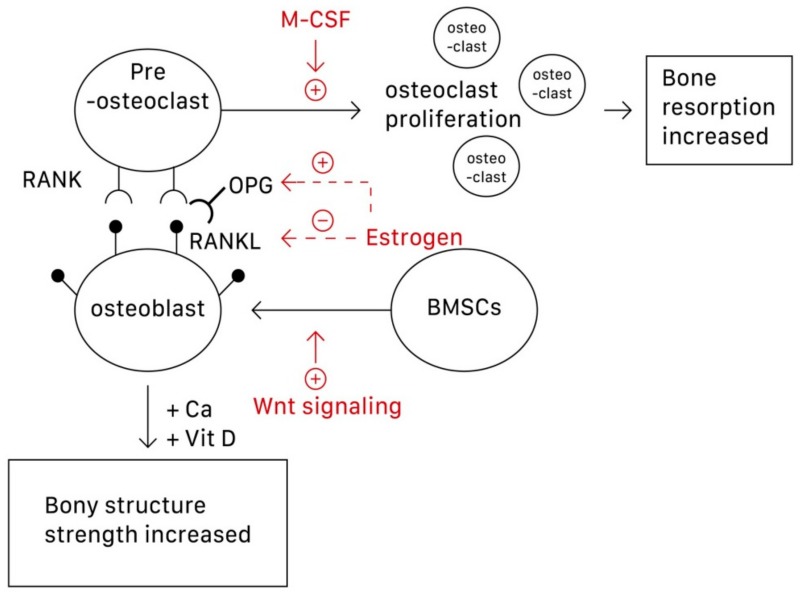
The pathways underlying the formation and action of osteoblasts, osteoclasts, and subsequent osteoporosis. M-CSF can promote the proliferation of osteoclasts; Wnt signaling can stimulate the differentiation of BMSCs into osteoblasts. Estrogen can both increase the secretion of OPG and decrease secretion of RANKL, thus preventing the combination of RANKL-RANK to activate osteoclastogenesis. BMSCs, bone marrow stromal cells; OPG, osteoprotegerin; RANKL, receptor activator of nuclear factor-κB (NF-κB) ligand).

Although osteoclasts are the main focus of osteoporosis therapy, osteoblasts have recently been established as potential targets [8]. Specifically, the interactions among the RANK/RANKL (receptor activator of nuclear factor-κB (NF-κB) ligand)/OPG (osteoprotegerin) system, Wnt/β-catenin signaling, and interleukin within osteoblasts and osteoclasts construct the bone remodeling cycle [8,9,10]. This is because osteoblasts can produce both RANKL and OPG. The function of OPG is to prevent RANKL from binding to RANK, which lies on the surface of osteoclasts and activates the proliferation of osteoclasts and bone resorption, thus inhibiting osteoclastogenesis. Wnt signaling regulates bone homeostasis by stimulating osteoblast differentiation and function and inhibiting osteoclast differentiation, mostly through indirect mechanisms via osteoblasts [8]. Wnt signaling can be classified into noncanonical (β-catenin-independent) and canonical (β-catenin-dependent) signaling pathways. In the latter, β-catenin accumulates in the cytoplasm and engages the nuclear area to express bone formation genes such as OPG and RANKL [9]. Estrogen is perhaps the most direct and effective approach to preventing and treating osteoporosis because it inhibits osteoclastic bone resorption. Osteoclasts require activation by two cytokines, namely M-CSF and RANKL, which are produced by BMSCs and osteoblasts, respectively. Estrogen suppresses osteoclasts by reducing the expression of RANKL in marrow cells and increases OPG secretion by osteoblasts, which inactivates RANK [10]. Estrogen thus decreases the secretion of RANKL and inactivates RANKL mediating osteoclastogenesis. In postmenopausal women, the estrogen level in the body decreases, dysregulating the RANKL/RANK/OPG system, and osteoclastogenesis then exceeds osteoblastogenesis [10]. Figure 1 shows the pathways underlying the actions of osteoblasts, osteoclasts, and subsequent osteoporosis.

Nutritional support, such as adequate calcium and vitamin D, as well as exercise are among the crucial cornerstones of the prevention of osteoporosis and are necessary during treatment with pharmacologic agents [1,11,12,13,14]. However, current evidence has shown that routine calcium and vitamin D supplements in healthy individuals are not needed. In patients with an adequate vitamin D status and a normal calcium intake, routine calcium and vitamin D supplements are not required in most people receiving treatment for osteoporosis, where routine use has not been shown to affect treatment efficacy [15,16]. Several new therapeutic agents are now available for the treatment of osteoporosis [3]. Although most have been found to significantly reduce the occurrence of vertebral fractures, controversy remains regarding the evidence for their nonvertebral or hip anti-fracture effects [1,3,8]. Nevertheless, physical modalities have been shown to have some beneficial effects in people with osteoporosis [17]. Based on a systematic survey of the current literature, this review provides evidence-based information for clinicians on the assessment of osteoporosis and the effects of treatment. Listed in the next section is a summary of current nonpharmacologic and nonsurgical treatments for osteoporosis.

## 2. Calcium and Vitamin D Supplementation

### 2.1. Calcium

Calcium is a vital element in human physiology. It plays a key role in muscle contraction, bone strength, nerve impulses and transmission, regulation of the heartbeat, and fluid balance within cells [1]. With regard to calcium deposition, calcium hydroxyapatite crystals add into the predominant type I collagen matrix in bone and increases bone strength [6]. By contrast, insufficient calcium intake causes a suboptimal bone mass peak and low bone mineralization, possibly leading to osteoporosis and fractures.

Because peak adult bone mass is achieved between age 30 and 35 years, elderly people and adolescents must maintain a higher calcium intake [12]. However, it is impossible to accurately identify the oral calcium amount that needs to be ingested as numerous factors affect the intestinal absorption of calcium, including age, sex, gonadal function, ethnic group, and the pattern of calcium intake.

Although calcium is physiologically necessary, a recent systematic review has shown that people in several Asian countries have an average dietary calcium intake of less than 500 mg/day [13]. People in African and South American countries also have a low calcium intake between 400 and 700 mg/day. Only people in Northern European countries have a calcium intake greater than 1000 mg/day [13]. The National Health and Nutrition Examination Survey 2003–2006 data in the United States show that dietary supplements provide an adequate intake of calcium for only 9% of the whole population [14].

According to the calcium allowances recommended by the WHO, based on North American and Western European data and the current recommendations of the European Union, Australia, Canada, United States, and the United Kingdom, an adequate intake of calcium for children aged seven to nine is approximately 400–700 mg per day. For pubertal and adolescent boys and girls, the recommended calcium intake is approximately 800–1300 mg per day. For men aged 19–65 and women from 19 years of age to the menopause, approximately 800–1000 mg per day of elemental calcium is recommended [8,12]. The National Osteoporosis Foundation advises men above 65 years and postmenopausal women to consume at least 1200 mg per day of elemental calcium [8]. As a treatment for osteoporosis, the Committee on Medical Aspects of Foot and Nutrition Policy (COMA) recommends taking more than 700 mg of calcium per day to maintain bone health.

The role played by calcium in reducing the risk of fractures is controversial. A nine-year follow-up of a prospective cohort study in Korea, involving 2158 men and 2153 women aged over 50, evaluated the association between a higher dietary calcium intake and the risk of fracture. The result showed that increased dietary calcium intake was not associated with any reduction in the risk of fracture among Korean women [18]. A systematic review of two randomized controlled trials (RCTs) and 44 cohort studies in 2015 concluded that increasing calcium intake from dietary sources cannot prevent fractures. In four RCTs (*n* = 44,505), the evidence showed that the effect of calcium supplements in preventing fractures is weak and inconsistent [19]. The European Society for Clinical and Economic Aspects of Osteoporosis, Osteoarthritis, and Musculoskeletal Diseases and the International Osteoporosis Foundation also concluded that current evidence reveals that supplementation with calcium alone cannot reduce the risk of fracture [12].

### 2.2. Vitamin D

Vitamin D is also an important factor in maintaining bone health. Following exposure of the skin to sunlight and UV B radiation, vitamin D3 is synthesized and subsequently converted into 25 hydroxyvitamin D in the liver by the enzyme 25-hydroxylase. It is then transported to the kidney, where it becomes 1,25-dihydroxycholecalciferol (also known as calcitriol), the active form of vitamin D in the human body [1]. Vitamin D can enhance the intestinal absorption of calcium, and it interacts with the parathyroid hormone to help maintain calcium homeostasis between the blood and bones. It can also regulate bone remodeling via binding with the vitamin D receptor (VDR) and initiating transcription of a VDR-associated gene. However, the direct effect of vitamin D on osteocytes remains unclear.

The dietary sources of vitamin D include salt-water oily fish and liver, egg yolks, margarine, some yogurts, cheeses, cereals, and vitamin D-fortified milk and orange juice. However, the major source of vitamin D3 for most humans is synthesis in the skin under the influence of UV light [1,15]. The prevalence of vitamin D deficiency has been reported to be higher in the elderly population. This might be due to insufficient skin exposure to sunlight and reduced efficacy of vitamin D synthesis. Indoor styles of living and clothing now result in low sun exposure [15,20]. UV irradiation, half the minimal erythematous dose, on 1000 cm^2^ of skin on the backs of elderly participants three times per week was found to be as effective as the oral intake of 400 IU vitamin D [20]. According to previous studies, the recommended daily oral dosage of vitamin D ranges from 400 to 2000 IU [1,8]. In a report by the National Osteoporosis Foundation, people older than 50 years are recommended to take at least 800–1000 IU of vitamin D daily because they are at greater risk of vitamin D deficiency [21]. However, a recent review revealed that supplements are better targeted based on clinical status to frail older people and possibly to people with dark skin living at higher latitudes [15]. Recent trials showed that vitamin D increases bone density when 25-hydroxyvitamin D levels are below 25–30 nmol/L. A daily dose of 400–800 IU (10–20 μg) is usually adequate [15]. Recent evidence has revealed that low dose vitamin D is safe, but high doses result in more falls and fractures [15,16]. Current evidence does not support the use of these supplements in healthy community-dwelling adults [15,16]. Vitamin D intoxication is one of the medical conditions and is typically observed when taking >10,000 IU of vitamin D per day for more than 5 months [22,23]. People who are at risk for hypercalciuria and hypercalcemia should monitor their 25(OH) D concentration more frequently.

However, whether direct vitamin D supplementation can increase BMD remains controversial. In 2019, a RCT was conducted to treat 400 people between 30 and 60 years of age for vitamin D deficiency with 50,000 IU per week for 8 weeks. The results showed that the prevalence of osteoporosis in the intervention group was significantly lower than that in the control group [24]. However, another prospective, randomized, double-blind, placebo-controlled, three-year clinical trial of vitamin D3 supplementation in 260 postmenopausal African American women showed no significant difference in BMD between placebo and vitamin D3 groups, although participants in the intervention group performed better in terms of gait speed and grip strength [25]. Moreover, another RCT published in 2019, comprising a three-year follow-up of 311 healthy men and women aged 55–70 years, concluded that a greater loss of BMD occurred with a higher intake of vitamin D (4000 IU per day or 10,000 IU per day, compared with 400 IU per day). Furthermore, no significant difference between the three groups in terms of bone strength was found [26]. The conflicting results and discrepancy among these three studies may be due to different age and race of participants, different dosage of vitamin D and study design [24,25,26]. In order to minimize study heterogeneity in future research, we urge standardization of the many variables involved in trials, such as age of participants and time of usage that may have crucial effect on the response. Dosage of vitamin D should be consistent across trials for better communication and prediction of therapeutic activity. Finally, bigger sample sizes are needed to increase the validity and generalizability of results.

### 2.3. Combined Use of Calcium and Vitamin D

Regarding the supplementation of calcium and vitamin D, an increase in dietary calcium should be considered first. When adequate dietary calcium intake cannot be achieved, calcium supplement tablets could be used [1]. Calcium carbonate is bioavailable when taken with a meal. Calcium citrate is recommended for individuals with a history of renal stones.

The best sources of calcium are dairy products including milk, yogurt and cheese. Calcium is also found in nuts, dark-green leafy vegetables, dried peas and beans. Vitamin D occurs naturally in only a few foods (salt-water oily fish, egg yolks, and liver,) and is also available in fortified foods (milk and milk products such as yogurts, cheeses and margarines), some juices, and breakfast cereals [14]. While the dietary sources of vitamin D are often insufficient to meet daily requirement, skin exposure to sunlight may be considered to provide a good resource of vitamin D by synthesis [20].

Although no consistent results have been found for the benefits of calcium or vitamin D supplements alone, a combination of calcium and vitamin D treatment has been shown to have some benefits in terms of reducing the incidence of fractures [27,28]. Several prospective, randomized, placebo-controlled trials have demonstrated the benefits of calcium and vitamin D. The National Osteoporosis Foundation published a meta-analysis in 2016 on calcium plus vitamin D supplementation and fracture prevention [27]. It included eight RCTs assessing calcium plus vitamin D supplementation versus a placebo on the incidence of total fracture. The enrolled calcium dosage was approximately 500 mg/day in one RCT study and approximately 1000–1200 mg/day in another [27]. The vitamin D dosage was approximately 800 IU/day in four RCT studies and 400 and 700 IU/day in the remaining two studies. The results showed a significant reduction of 15% in the risk of all fractures and a greater reduction of 30% in the risk of hip fractures [27]. Another large study in 2010, published by the DIPART (Vitamin D Individual Patient Analysis of Randomized Trials) Group, recruited seven RCTs of either vitamin D with calcium or vitamin D alone, with fractures as an outcome. This study involved a total of 68,517 participants. The results revealed that vitamin D given alone in doses of 10–20 μg was unable to prevent fractures. However, calcium and vitamin D together led to a decrease in hip fractures and total fractures, and probably also vertebral fractures [28]. Conversely, the Women’s Health Initiative Calcium and Vitamin D trial (WHI CaD) found no association between combined supplements and height loss in 36,282 healthy postmenopausal women, compared with a placebo. In this study, 1000 mg of elemental calcium in the form of calcium carbonate with 400 IU of daily vitamin D and a placebo were compared [29]. Recently, the major trials in community-dwelling individuals have also not demonstrated fracture prevention with a combination of calcium and vitamin D [15,16]. Nevertheless, the results of a large study in vitamin D-deficient nursing home residents indicated a reduced fracture incidence [15]. These conflicting results are likely to be due to differences in dosage of supplements, patient populations, and study design [15,16,28,29]. For individuals living in residential institutions, they are more likely to have osteoporosis because of their poorer mobility, infrequent sun exposure, and poorer diet [16]. For these reasons it is possible that older people living in residential care institutions may benefit from calcium and vitamin D supplements. In summary, benefits of calcium and vitamin D supplementation may differ between people living in the community and people living in residential institutions [16].

In summary, there is currently no recommendation for the individual use of calcium or vitamin D supplementation to reduce the risk of fracture. However, a combination of calcium and vitamin D supplementation may have some benefits in preventing fractures in the future for specific populations.

## 3. Lifestyle

A large number of risk factors for osteoporotic fracture have been identified by epidemiological studies. Osteoporotic fractures commonly involve the vertebral body, hip joints, or wrists [4]. Among these fractures, hip fractures are life-threatening. Approximately 5–20% of patients die within a year, and 50% of survivors have difficulty functioning and thus need extra care [30].

However, some of the identified risk factors for osteoporotic fracture are modifiable. Maintaining an ideal body weight, quitting smoking, and performing regular weight-bearing exercise are beneficial for bone health. A history of smoking is associated with a higher risk of fractures than nonsmokers, but a lower risk than current smokers [31]. No effective and safe methods were provided in the clinical field that could help osteoporosis patients to recover. Low calcium intake, high sodium intake, and excessive alcohol and cola consumption reduce bone mass [32]. The consumption of 20 g or more ethanol per day also shows a dose–effect relating to an increasing fracture rate. Cola containing high phosphoric acid eliminates dietary calcium in the intestinal tract and bone mineral is dissolved to neutralize acid [33]. Administration of glucocorticoids and anticonvulsants also adversely affect bone health.

## 4. Fall Prevention

Osteoporosis, falling, and fragility-related fractures have been identified as a serious global public health issue. According to the Web-based Injury Statistics Query and Reporting System run by the Centers for Disease Control and Prevention, National Center, falls are the leading cause of both fatal and nonfatal injuries of the elderly. According to some regional or national reports, one in every three older adults living in the community experiences at least one fall every year. For those living in residential care facilities or nursing homes, the annual risk of a fall is more than 50% [34]. Between 71.6% and 92.4% of fractures in community-dwelling older populations (age >50 years) are osteoporotic [35]. The most frequent osteoporotic fracture site was the hip, followed by the humerus and then the wrist [36]. A total of 2.3 million nonfatal fall injuries among older adults were treated in emergency departments in 2010, and more than 662,000 of these patients were hospitalized. The direct medical expenditure for nonfatal fall-related fractures was approximately $12 billion in 2013 [35]. Many people who fall, even if they are not injured, develop a fear of falls and tend to limit their activities, resulting in reduced mobility and a loss of physical fitness and bone mass. Fear of falling has been identified as a risk factor for falls [35,37,38].

Fall prevention needs comprehensive management, including nutrition, prescription medicine, changes in living behavior, exercise plans, and so on. Simple physical training [37,38], such as Tai Chi, can improve muscle strength, balance behavior, and walking speed [39,40,41]. For those living in residential care facilities or nursing homes who have a cognitive impairment, hip protectors may have some immediate benefit in reducing the risk of fracture [42].

Several hazardous factors in the living environment should be removed to prevent falls [43,44]. Handrails mounted on the wall, stairs, and a light environment are recommended. Slippery carpets should be fixed to the ground. A floor surface with low resistance should be avoided. For outdoor conditions, rubber soles with a relatively high coefficient of friction should be worn to prevent falls as a result of slipping. Additionally, reducing a medicine dosage that might jeopardize balance behavior and cognition status will also lower the chance of falling.

## 5. Exercise

Exercise can improve or maintain bone mass in all ages and numerous research studies have demonstrated that exercise can improve physical function, quality of life, pain, and vitality in osteoporotic and osteopenia postmenopausal women [45]. These specific exercises should be dynamic, exceed a threshold intensity and strain frequency, impose an unusual loading pattern on the bones, and be supported by unlimited nutrient energy [46].

The reason why osteoporosis can be avoided or delayed by conducting exercise is that the mechanical stress generated during the exercise can cause certain deformation of bone tissue, which then stimulates the osteoclasts and osteoblasts. Related research indicates that mechanical stress is transformed into a signal that induces the synthesis of DNA, and eventually, increases BMD. In brief, bone tissue deforms upon exercise, and thus the mechanosensors located throughout the cells, such as stretch-activated ion channels and integrins, change their original conformation to trigger a signaling cascade to provide an appropriate biochemical response (such as osteogenesis and bone accretion at the site of deformation) [47]. As described in the Introduction section, exercise can activate the Wnt/β-catenin signaling pathway to initiate osteogenesis and bone formation, either by direct stimulation of the bone transcription factor RUNX2 or by cross-talking with PTH or morphogenetic proteins (BMPs) signaling pathways [47]. Circulating PTH, generated from physical activity, leads to downregulation of sclerostin (an anti-anabolic bone protein) in osteocytes, which was accompanied by significant upregulation of fibroblast growth factor-23 (FGF-23) expression, a growth factor governing phosphatase homoeostasis and vitamin D metabolism [47]. Additionally, exercise leads to an increase in OPG/RANKL ratio and consequent inhibition of osteoclast differentiation, promotion of osteoblast differentiation and bone formation by aforementioned mechanism, as shown in Figure 1 and the Introduction section [48].

Bone tissue can also regulate the reconstructing process to bear exercise loading. The rate of bone formation with constant exercise is higher than that without; therefore, a dynamic equivalence between BMD and exercise intensity is reached [47]. For women with amenorrhea, demineralization can be restrained through constant exercise. By contrast, for bedridden patients or the elderly people, a lack of exercise can lower BMD and therefore increase the probability of developing osteoporosis.

Strengthening muscles, balance training, and coordination exercises are strongly recommended. The impulse force from the ground during open kinetic exercises, such as jogging, speed walking, and drop jumping, along with reacting forces due to muscle contraction, stimulate bone growth and increase BMD [47]. Stimulation of bone tissue as a result of high impulse exercise is considerably stronger than in lower impulse exercise.

Resistive exercise is a form of physical activity that is designed to improve muscular fitness by exercising a muscle or a muscle group against external resistance. Resistive exercise can be accomplished with traditional free weights and dumbbells, weight machines, body weight, elastic tubing, medicine balls, or even common household products like milk jugs filled with sand or soup cans [49]. It helps maintain functional capacity, prevent and recover from injuries, and improve sports performance [50].

Resistive exercise training can also increase BMD, prevent osteoporosis, lower the risk of falling, strengthen muscles, and improve the ability to maintain balance [49,51]. A related study indicates that resistive exercise three to four times a week can effectively prevent osteoporosis. In women, during or after menopause, intensive resistive-aerobic exercise or progressive resistive exercise increases BMD by 1–4% each year. More effective stimulation can be achieved by fast lifting than slow lifting, long-term exercise than short-period exercise, and heavy lifting than light lifting [52].

Training targets are specifically recommended to raise the BMD of areas at high risk of osteoporosis, such as the femur, carpus, and thoracolumbar spine. Previous studies have suggested that BMD of the femur can be increased substantially by walking [37]. Light walking for 4 h each week could significantly prevent hip fracture. Several previous studies have shown that slightly-heavy-loading aerobic exercise, hip impulse training, and resistive training can effectively increase the BMD of the lumbar spine. Shigeta and Goto [53] found that the BMD of women before menopause continuously increases as a result of raising their level of metabolism. For women after the menopause, raising their level of metabolism prevented bones from developing osteoporosis, even though the BMD was not significantly increased [17,54,55].

An appropriate level of exercise could also help prevent osteoporosis. However, excessive exercise might jeopardize the growth of the bone and interrupt the endocrine system, mitochondria, pituitary gland, or ovarian functioning. These might lead to endocrine disorder, menstrual disorder, and ultimately, osteoporosis. A related survey found that athletes who constantly perform excessive exercise have lower BMD than nonathletes [56,57]. To reduce the risk of overtraining for high-performing athletes, a dramatic increase in volume should be avoided. It is recommended that a 2–10% increase in the load be applied when the individual can comfortably perform the current workload for one to two repetitions over the desired number on two consecutive training sessions [49]. For adults, the American College of Sports Medicine recommends weight-bearing endurance and plyometric exercise three to five times per week, and resistive exercise of moderate to high loading two to three times per week for a total of 30 to 60 min per day [58].

In summary, high intensity strength training and low impact weight-bearing exercise form part of a core management strategy for osteoporosis. For elderly people with a balance disability, exercise, such as muscle strengthening and balance training, should be conducted under the strict supervision of experts. For patients suffering from a fracture, a cautious strategy and a targeted exercise method should be adopted. People suffering from osteoporosis should avoid performing an impulsive exercise that might cause serious injuries. For people with potential osteoporosis, excessive motion, such as bending or heavy lifting, should be avoided.

## 6. Physical Modalities

Physical modalities, including low-intensity pulsed ultrasound (LIUS) and electrical FDA approved stimulation, can be used to accelerate bone repair [59].

### 6.1. Low-Intensity Pulsed Ultrasound

Ultrasound is a high-frequency nonaudible acoustic pressure wave traveling in the form of mechanical energy that can be directed at biological tissues to exert a mechanical stimulus. In vitro cellular studies have found that LIUS can regulate bone cells; enhancing osteoblast formation and function while suppressing osteoclast formation and function [60,61,62]. Using in vivo animal models, LIUS has been shown to increase bone mineral content and enhance the mechanical properties of the healing callus and bone bridging. These result in greater bone marrow density and mechanical strength in the LIUS-treated area than in controls [63,64,65,66]. In humans, several clinical trials have also shown that LIUS can accelerate bone healing in patients with distal radius fractures, tibial fractures, and delayed union of osteotomized fibulas [67,68,69], and reduce union time in fresh fractures [68,69]. The exact mechanism of action remains uncertain. Most subjects in treatment groups received LIUS with a 200 μs burst width of 1.5 MHz frequency sine waves, 1 kHz pulse repetition, and a spatial temporal average intensity of 30 mW/cm^2^ for 20 min daily.

However, there are conflicting results on the effects of LIUS on osteoporotic bones. Carvalho and Cliquet [70] and Perry et al. [71] found increased bone formation rate and enhanced microarchitecture in LIUS-treated groups, compared with control groups. Conversely, Warden et al. observed that LIUS did not have any positive skeletal impact on spinal cord injury (SCI), and concluded that LIUS was not a beneficial intervention for SCI-induced osteoporosis because of its inability to penetrate the intact outer cortex of the bone [72]. The reasons for controversy over the effects on osteoporotic bones may be attributable to uncontrollable variables and several intrinsic and extrinsic limitations, such as heterogeneous bone architecture and individual variation [70,71,72]. LIUS application is also restricted to small body regions because these waves travel as a relatively focused beam (typical effective radiating area = 5 cm^2^). Therefore, LIUS cannot provide any benefits for systemic metabolic bone diseases.

Delayed union is a process of union occurring by late intramedullary callus, the periosteal response having ceased before union was achieved. As an end point, delayed union is represented by evident cessation of periosteal new bone formation before union has been achieved, likely to be reflected by a bending stiffness of less than 7 N-m per degree by 20 weeks [73]. Nonunion is a process of scar formation in which the rate of endosteal and periosteal osteogenesis is zero or low, being outweighed by bone resorption. As an end point, nonunion is evidenced by cessation of periosteal and endosteal new bone formation, with sclerosis of the medullary canal observed at the fracture surfaces [73]. On the basis of current evidence, LIUS is a relatively safe and potentially efficacious treatment option to promote the healing of nonunion and delayed union fractures.

### 6.2. Electrical Stimulation

Electrical stimulation of bone fusion is a technique that has been used for three decades. Three types of electrical stimulation technologies been approved for clinical use: direct current (DC); inductive coupling (IC) such as pulsed electromagnetic fields (PEMF) and combined magnetic fields (CMF); and capacitive coupling (CC) [74,75,76,77,78]. This section focuses on noninvasive IC and CC technologies because DC technology requires surgical implantation of the device. PEMF and CMF consist of external current-carrying coils that produce a magnetic field, which induces a secondary electric field at the fusion site. The CC device consists of electrodes applied to conductive gel on the skin that produce an electric field at the fusion site [74,75,76,77,78].

Bone is a piezoelectric material. Thus, when it is mechanically strained, electrical potentials are generated. The electric fields generated by electrical stimulation devices up-regulate a number of growth factors involved in normal bone healing. The theoretical and biological mechanism is not fully understood. A dose–response effect, where cell proliferation increases as treatment time increases, was observed for bone cells treated with IC and CC. CC stimulation elicited a greater response than IC stimulation [74]. For postmenopausal women with multiple fractures and chronic back pain, a larger proportion of an active group treated with CC stimulation were able to discontinue the use of NSAID to eliminate pain than that of a control group [75]. Several studies have found that electrical stimulation significantly promoted osteogenesis in osteoporotic or osteoporosis-prone women [76,77] and bilateral ovariectomy rats [78]. No negative side effects have been reported with IC and CC for bone growth over the past twenty years [75,79]. Electric stimulation technologies have been used clinically as a potentially effective adjunctive treatment for fracture healing, and to inhibit or reverse osteopenia. In patients who are pregnant or have cancer or pacemakers, these electrical stimulation treatments should be avoided [79]. Because the safety of other physical modalities for pregnant women or those who have cancer or pacemakers has not been established, physical modalities including low-intensity pulsed ultrasound and whole-body vibration were not recommended for such patients in whom electrical stimulation cannot be used. Alternatively, light exercise is usually appropriate and may be considered to be done according to the general condition of every patient.

### 6.3. Whole-Body Vibration

Whole-body vibration has been widely applied in environmental adjustment for astronauts, physical training, and clinical treatment for the last 20 years. The effects of whole-body vibration depend on vibration mode, amplitude, and frequency. Different selections can have varying effects on different target groups [80].

Vibration stimulation alters the pressure within the bone channel, leading to a piezoelectric response, increasing the sediment rate of newly grown bone and minerals, and eventually, accelerating the healing process [81]. A vibration of 30 Hz for a month on the bones of turkeys increases the sediment rate of newly grown bone and minerals [82]. Clinical research by Gusi and Raimundo [51] showed that vibration training could effectively increase the BMD and balance ability of women after menopause [51]. Moreover, vibration training could lower the risk of developing osteoporosis. Vibration training of 50 Hz for 30 min each day was also found to prevent osteoporosis in rats with removed ovaries [83]. However, for normal rats with healthy ovaries, vibration training did not work [83]. In the study, the explanation for the result is that vibration suppresses bone turnover, which is increased after menopause or oophorectomies. From the mechanical point of view, the lack of estrogen after menopause or oophorectomies will alter bone remodeling, accelerating bone turnover and resulting in an eventual bone loss. In contrast, vibration training did not work for normal rats with healthy ovaries because that the bone turnover is not increased in this stage [83].

Strength and rapid force can be increased by vibration training. Vibration force induces the tonic vibration reflex of the muscle and lowers the inhibition reaction of the Golgi tendon organ, causing a reflexing reaction by activating the muscle spindle [84]. Vibration training on female athletes, at a frequency of 26 Hz and amplitude of 10 mm for ten min, increased the strength of the muscle for a short period [85]. Vibration training on the elderly, at a high frequency for 2 months, was found to significantly improve their strength [86].

Bones are relatively sensitive to high frequency and low amplitude. Therefore, they grow effectively under such conditions. The amplitude of vibration stimulation can be set between 1 and 10 mm and the frequency of vibration stimulation can be set between 10 and 80 Hz [87]. Kiiski et al. proposed that a frequency of 10–60 Hz and an amplitude of 0.05–5 mm should be chosen for elderly people and postmenopausal women to stimulate the synthesis and metabolism of trabecular bone and slow the process of osteoporosis. When this was applied, BMD clearly increased [88]. However, a RCT conducted by Kiel et al. [89] revealed different results. The study randomized 174 senior men and women (89 active, 85 placebo) from 16 independent living communities, who had T-scores –1 to –2.5, were not taking bone active drugs and had no diseases affecting the skeleton. All of the participants received daily calcium (1000 mg) and vitamin D (800 IU), and the participants in the active group underwent 10 min of daily whole-body vibration (0.3 g at 37 Hz) [89]. The authors found that daily whole-body vibration in older adults did not demonstrate evidence of significant beneficial effects on volumetric BMD or bone biomarkers. The beneficial effects of whole-body vibration observed in previous studies of younger women may not occur to the same extent in elderly individuals [89].

Vibration treatment can not only improve the behavior of muscles and bone but can also be beneficial for pain management and balance ability. The level of pain experienced by young participants decreased significantly after performing low frequency vibration treatment [90]. Van Nesbet et al. performed whole-body vibration treatment on patients with a walking disability and found that it could effectively improve their ability to stand upright and shift their weight [91]. The longer the vibration treatment, the more effective it was in raising BMD. Gusi and Raimundo [51] stated that, compared with the use of 24-week vibration treatment by Verschueren and Roelants [92], the 32-week vibration treatment was more effective in raising the BMD of hip bones. However, the optimal duration of vibration exposure remains unknown.

For people who are unwell or unable to perform strenuous and time-consuming conventional exercise, whole-body vibration might be a viable alternative to improve bone health. It is also viewed as a potentially safe, low impact substitute for current modalities to counteract muscle and bone deterioration in mobility-limited individuals without the risks associated with high impact exercise [51,81,82,83,84,85,86,87,88]. However, some potential risks involved in vibration treatment have been indicated. For instance, experts have suggested that prolonged vibration treatment could lead to temporary dizziness and impairment of consciousness [93,94,95]. Moreover, prolonged treatment duration with excessive amplitude can induce chronic fatigue and damage and degeneration of tissue. Whole-body vibration should therefore be applied in a safe environment and with continuous monitoring to prevent discomfort and any physical damage.

## 7. Summary

Effective fracture prevention by reducing loss of bone mass is the primary goal for physicians treating people with osteoporosis. Other than pharmacologic agents, lifestyle factors, nutritional support, fall prevention strategies, exercise, and physical modalities can be used to treat osteoporosis or prevent further osteoporotic fracture. Each factor, or a combination of these, can benefit people with osteoporosis and should be implemented after detailed discussion with patients. This review article explored current medical knowledge on osteoporosis and its nonpharmacologic and nonsurgical treatments. However, numerous issues regarding osteoporosis and its treatment remain unknown or controversial and warrant further investigation.
